# 2357. Risk Factors for COVID-19 Infection and Outcomes in People Living with HIV

**DOI:** 10.1093/ofid/ofac492.164

**Published:** 2022-12-15

**Authors:** John J Hanna, Liyu B Geresu, Marlon Diaz, Madison Pickering, Julia A Casazza, Milan Ho, Heather Lanier, A L exander P Radunsky, Sameh N Saleh, Zachary M Most, Trish M Perl, Robert W Turer, Christoph U Lehmann, Jeremy Y Chow, Richard J Medford

**Affiliations:** University of Texas Southwestern, Dallas, Texas; University of Texas Southwestern, Dallas, Texas; University of Texas Southwestern Medical Center, Dallas, Texas; University of Chicago, Chicago, Illinois; University of Texas Southwestern Medical Center, Dallas, Texas; University of Texas Southwestern Medical Center, Dallas, Texas; University of Texas Southwestern, Dallas, Texas; Ut Southwestern, Dallas, Texas; University of Texas Southwestern, Dallas, Texas; University of Texas at Southwestern Medical Center, Dallas, Texas; University of Texas Southwestern Medical Center, Dallas, Texas; University of Texas Southwestern Medical Center, Dallas, Texas; University of Texas Southwestern Medical Center, Dallas, Texas; UT Southwestern, Dallas, Texas; University of Texas Southwestern Medical Center, Dallas, Texas

## Abstract

**Background:**

As the risk for concomitant COVID-19 infection in people living with HIV (PLHIV) remains largely unknown, we explored a large national database to identify risk factors for COVID-19 infection among PLHIV.

**Methods:**

Using the COVID-19 OPTUM de-identified national multicenter database, we identified 29,393 PLHIV with either a positive HIV test or documented HIV ICD9/10 codes. Using a multiple logistic regression model, we compared risk factors among PLHIV, who tested positive for COVID-19 (5,134) and those who tested negative (24,259) from January 20, 2020, to January 20, 2022. We then compared secondary outcomes including hospitalization, Intensive Care Unit (ICU) stay, and death within 30 days of test among the 2 cohorts, adjusting for COVID-19 positivity and covariates. We adjusted all models for the following covariates: age, gender, race, ethnicity, U.S. region, insurance type, adjusted Charlson Comorbidity Index (CCI), Body Mass Index (BMI), and smoking status.

**Results:**

Among PLHIV, factors associated with higher odds for acquiring COVID-19 (Figure 1) included lower age (compared to age group 18–49, age groups 50–64 and >65 were associated with odds ratios (OR) of 0.8 and 0.75, P= 0.001), female gender (compared to males, OR 1.06, P= 0.07), Hispanic White ethnicity/race (OR 2.75, P= 0.001), Asian (OR 1.35, P= 0.04), and African American (OR 1.23, P= 0.001) [compared to non-Hispanic White], living in the U.S. South (compared to the Northeast, OR 2.18, P= 0.001), being uninsured (compared to commercial insurance, OR 1.46, P= 0.001), higher CCI (OR 1.025, P= 0.001), higher BMI category (compared to having BMI< 30, Obesity category 1 or 2, OR 1.2 and obesity category 3, OR 1.34, P= 0.001), and noncurrent smoking status (compared to current smoker, OR 1.46, P= 0.001). Compared to PLHIV who tested negative for COVID-19, PLHIV who tested positive, had an OR 1.01 for hospitalization (P = 0.79), 1.03 for ICU stay (P=0.73), and 1.47 for death (P=0.001).

**Conclusion:**

Our study found that among PLHIV, being Hispanic, living in the South, lacking insurance, having higher BMI, and higher CCI scores were associated with increased odds of testing positive for COVID-19. PLHIV who tested positive for COVID-19 had higher odds of death.

**Disclosures:**

**Christoph U. Lehmann, MD**, Celanese: Stocks/Bonds|Markel: Stocks/Bonds|Springer: Honoraria|UTSW: Employee **Jeremy Y. Chow, M.D., M.S.**, Gilead Sciences: Grant/Research Support.

**Figure 1.**
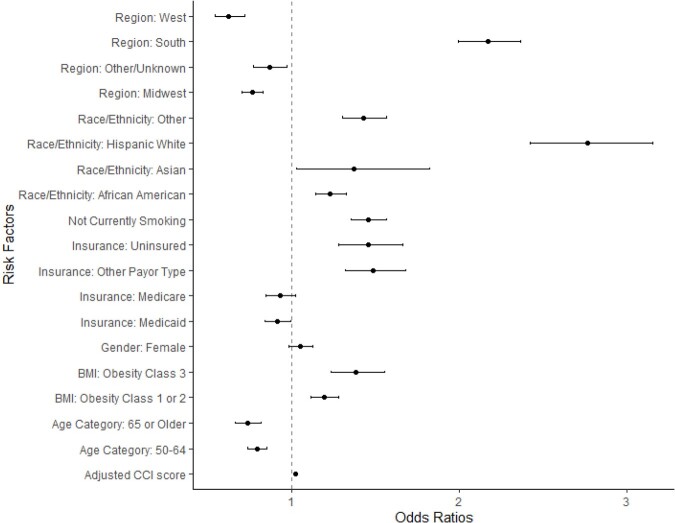
Risk Factors for COVID-19 Infection in PLHIV

Odds Ratio References Include:

Age Category: <50, BMI: Non-Obese, Gender: Male, Insurance: Commercial, Smoking: Currently Smoking, Race/Ethnicity: Non-Hispanic White, Region: Northeast

